# Multimodal monitoring intracranial pressure by invasive and noninvasive means

**DOI:** 10.1038/s41598-023-45834-5

**Published:** 2023-10-27

**Authors:** Fabiano Moulin de Moraes, Erica Navarro Borba Adissy, Eva Rocha, Felipe Chaves Duarte Barros, Flávio Geraldo Rezende Freitas, Maramelia Miranda, Raul Alberto Valiente, João Brainer Clares de Andrade, Feres Eduardo Aparecido Chaddad-Neto, Gisele Sampaio Silva

**Affiliations:** Neurology and Neurosurgery Department, Federal University of São Paulo, São Paulo, Brazil

**Keywords:** Neurology, Medical imaging, Therapeutics

## Abstract

Although the placement of an intraventricular catheter remains the gold standard method for the diagnosis of intracranial hypertension (ICH), the technique has several limitations including but not limited to its invasiveness. Current noninvasive methods, however, still lack robust evidence to support their clinical use. We aimed to estimate, as an exploratory hypothesis generating analysis, the discriminative power of four noninvasive methods to diagnose ICH. We prospectively collected data from adult intensive care unit (ICU) patients with subarachnoid hemorrhage (SAH), intraparenchymal hemorrhage (IPH), and ischemic stroke (IS) in whom invasive intracranial pressure (ICP) monitoring had been placed. Measures were simultaneously collected from the following noninvasive methods: optic nerve sheath diameter (ONSD), pulsatility index (PI) using transcranial Doppler (TCD), a 5-point visual scale designed for brain Computed Tomography (CT), and two parameters (time-to-peak [TTP] and P2/P1 ratio) of a noninvasive ICP wave morphology monitor (Brain4Care[B4c]). ICH was defined as a sustained ICP > 20 mmHg for at least 5 min. We studied 18 patients (SAH = 14; ICH = 3; IS = 1) on 60 occasions with a mean age of 52 ± 14.3 years. All methods were recorded simultaneously, except for the CT, which was performed within 24 h of the other methods. The median ICP was 13 [9.8–16.2] mmHg, and intracranial hypertension was present on 18 occasions (30%). Median values from the noninvasive techniques were ONSD 4.9 [4.40–5.41] mm, PI 1.22 [1.04–1.43], CT scale 3 points [IQR: 3.0], P2/P1 ratio 1.16 [1.09–1.23], and TTP 0.215 [0.193–0.237]. There was a significant statistical correlation between all the noninvasive techniques and invasive ICP (ONSD, *r* = 0.29; PI, *r* = 0.62; CT, *r* = 0.21; P2/P1 ratio, *r* = 0.35; TTP, *r* = 0.35, *p* < 0.001 for all comparisons). The area under the curve (AUC) to estimate intracranial hypertension was 0.69 [CIs = 0.62–0.78] for the ONSD, 0.75 [95% CIs 0.69–0.83] for the PI, 0.64 [95%Cis 0.59–069] for CT, 0.79 [95% CIs 0.72–0.93] for P2/P1 ratio, and 0.69 [95% CIs 0.60–0.74] for TTP. When the various techniques were combined, an AUC of 0.86 [0.76–0.93]) was obtained. The best pair of methods was the TCD and B4cth an AUC of 0.80 (0.72–0.88). Noninvasive technique measurements correlate with ICP and have an acceptable discrimination ability in diagnosing ICH. The multimodal combination of PI (TCD) and wave morphology monitor may improve the ability of the noninvasive methods to diagnose ICH. The observed variability in non-invasive ICP estimations underscores the need for comprehensive investigations to elucidate the optimal method-application alignment across distinct clinical scenarios.

## Introduction

Intracranial hypertension (ICH) is a frequent and critical complication after acute brain injury^1,2^. Intracranial pressure (ICP) measurement is considered the standard of care to guide therapy in patients with potential ICH^3^, although there is no definitive evidence supporting the usefulness of ICP monitoring to improve patient outcomes^4^.

Despite several limitations of the invasive method for monitoring ICP (ICPi)^5^, a noninvasive method (ICPni) that meets all the requirements to replace it is still not available^6^. Recently, either the association of noninvasive methods^7^ or the creation of new ICPni monitoring techniques has renewed the interest in these technologies^8–10^.

In this context, in this study we evaluated the correlation between ICPi and four noninvasive methods: a noninvasive waveform pulse morphology (P2/P1 ratio and Time-To-Peak [TTP) (Brain4care^9,11^ technology), the optic nerve sheath diameter (ONSD)^12^, pulsatility index (PI) by transcranial Doppler (TCD)^13^, and a five-item visual scale assessed by brain computed tomography (CT)^6,14^. Additionally, as an exploratory hypothesis generating analysis, we assessed the discriminant ability of these noninvasive methods to detect ICH.

## Methods

We conducted a prospective study of patients with acute ischemic (IS) or hemorrhagic stroke (HS) requiring ICPi monitoring due to the risk of ICH. We included patients admitted to a neurocritical care unit (neuro-ICU) of a tertiary hospital, aged 18 years or older, diagnosed at admission with IS or HS (subarachnoid and intraparenchymal hemorrhage) who required ICPi monitoring with the placement of external ventricular drainage (EVD). We excluded patients with chronic neurological diseases (demyelinating or neuromuscular disease), past medical history of diseases associated with high ICP (e.g., chronic hydrocephalus, benign intracranial hypertension), those under ICP monitoring other than intraventricular (e.g., subdural or epidural), or with suspected brain death. There was no direct public or private funding for this study, except for the availability of the device and the technical support given by Brain4care Inc. This study protocol was approved by our local Ethics Committee and registered at clinicaltrials.gov under number NCT05121155. All patients’ legal representative signed an informed consent form.

### Patient monitoring

All patients received the standard ICU continuous hemodynamic monitoring, which consisted of invasive blood pressure assessment through the radial artery, continuous electrocardiography, and pulse oximeter. Except for the CT scan, all tests were performed simultaneously, at least five minutes after EVD closure. ICP was assessed through a catheter inserted into the ventricle and connected to a pressure transducer placed at the level of the external auditory meatus and connected to a drainage system (Codman, Johnson and Johnson Medical Ltd., Raynham, MA, USA). Blood flow velocities were measured using TCD (DWL, Doppler BOXx, Compumedix, Germany) by a selected group of experienced certified operators. Insonation was standardized by positioning a 2 MHz pulsed low-frequency ultrasound probe in the temporal window for insonation of the M1 and M2 portions of the middle cerebral artery (MCA) at a depth of 45–55 mm bilaterally to measure the pulsatility index (PI), with one measurement on each side. PI was calculated automatically using the formula:

$$PI=\frac{\mathrm{Systolic \,Flow\, Velocity }\left(\mathrm{sys \,FV}\right)-\mathrm{Diastolic\, Flow\, Velocity }(\mathrm{dias\, FV})}{\mathrm{Mean\, Flow\, Velocity }(\mathrm{mFV})}$$

Optic nerve sheath ultrasound (ONSUS) was performed exclusively by the principal investigator using an US device with a 13–6 MHz linear probe with ocular presets (Samsung Mysono U6, EUA). Three measurements were made on each side (six measurements per patient). The final sheath value was the average of these six measurements. The probe was leaned against the upper eyelid with the eyes closed and caudally and medially angled until the optic nerve was visualized as a linear hypoechoic structure with well-defined margins posterior to the eyeball. The probe was never leaned against the patient’s cornea or conjunctiva. All images were collected on an axial/transverse axis for uniformity. The sheath was measured 3 mm behind the retina.

All CT measurements were made within 24 h of the other methods and assessed by certified neuroradiologists to detect the ICH signs described in Table 1. CT readers were blinded to ICPi measurements.

**Table 1:** CT findings predictive of Intracranial Hypertension.

1 Diffuse sulci effacement
2 Obliteration of basal cisterns
3 Hydrocephalus defined as:
a Both temporal horns with more than 2 mm and/or
b Evans Index > 0.3*
4 Midline shift > 5 mm
5 Transtentorial or uncal herniation

Adapted from Rajajee V., et al.^9^ CT: Computerized Tomography. *The Evans index is the ratio of the maximum width of the frontal horns of the lateral ventricles to the maximum internal diameter of the skull at the same level on axial sections of CT or MRI images.

**Figure 1:**
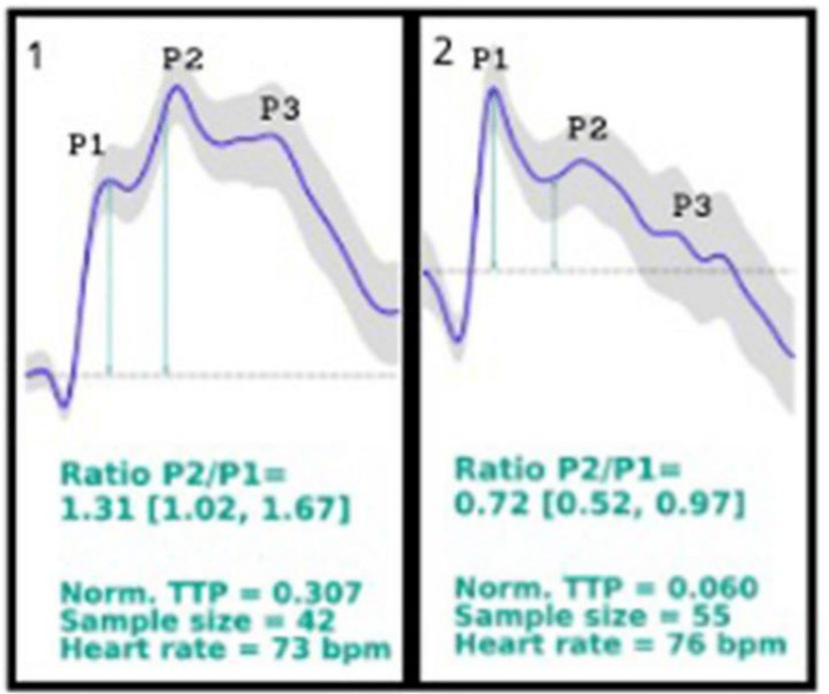
Waveform analysis of the Brain4care Monitor. (**A**): normal waveform (P1 > P2 and short TTP). (**B**): abnormal waveform (P2 > P1 and longer TTP). P2/P1 ratio: the ratio between the amplitudes of peaks P2 and P1; TTP: time to peak, defined as the time, from the start of the pulse, at which the ICP waveform reaches its highest peak. The shaded line represents the confidence interval of the 1-min monitoring.

The Braincare sensor (Braincare Monitor 2000 and Braincare ICPNI sensor [BcSsNI2000], Braincare Corp, USA) was positioned on the patient’s scalp without shaving, surgical incision, or trepanation after EVD closure. The Braincare Analytical System acquired ICP wave morphology through a strain sensor that, when in contact with the patient’s skin on the skull, can detect and monitor subtle bone deformations caused by ICP changes. These ICP oscillations generate pulse waves corresponding to the ICPi monitoring^9,11^. The pulse waveform was monitored throughout the session time. The device digitized, filtered, and amplified all recordings, which were later transferred to a computer for analysis. Brain4care® monitor results were accessed by professionals blinded to the results of the other noninvasive methods and the absolute ICPi value. Patients were monitored for at least 30 min. The ICPni waveform parameters calculated were the P2/P1 ratio and the time-to-peak (TTP). Calculations were performed using the B4c morphology after artifact exclusion. The mean pulse wave was used to calculate P1 and P2 peak amplitudes, with the peak arterial pressure wave being used to define P1 in case of disagreement^15^. The amplitude was obtained by detecting the highest point of these peaks and subtracting the initial ICPni wave value. P2/P1 ratio was calculated by dividing the amplitude of these two points. TTP is the time between wave onset and its maximum peak. The averaged values of P2/P1 and TTP were obtained from the entire recording (Fig. 1).

### Data analysis

Qualitative variables were expressed as absolute (n) and relative (%) frequencies, and quantitative variables were expressed as mean ± standard deviation, medians, minimum, and maximum values.

Repeated measures correlation (rmcorr) was used to assess the relationship between noninvasive methods and the absolute ICP value^16^. The intraclass correlation coefficient (ICC) was used to analyze the agreement between sides in the TCD and the ONSD measurements. Receiver operator characteristic (ROC) curves for repeated measures were constructed for each method to define the cutoff points of the noninvasive techniques, considering the presence or absence of ICH (mean ICP greater than 20 mmHg) as the reference standard^17^. The area under the curve (AUC) was used to describe the discriminatory power of the methods, and the Youden index was used to define the cutoff point for each method. Binary logistic regression models for repeated measures using generalized estimated equations were used to analyze how these methods related to ICH jointly. The results were presented as odds ratio (OR) and their respective 95% confidence intervals (CI95%). ICH was considered the dependent variable, and noninvasive methods as the independent variables. Statistical analysis was performed using the JAMOVI software (version 1.8.1.).

### Ethics approval and consent to participate

This study protocol was approved by our local Ethics Committee at https://plataformabrasil.saude.gov.br under number 03843118.0.0000.5505 and registered at clinicaltrials.gov under number NCT05121155. All patients’ legal representative signed an informed consent form. The research performed in accordance with the Declaration of Helsinki.

## Results

### Population description

**Figure 2:**
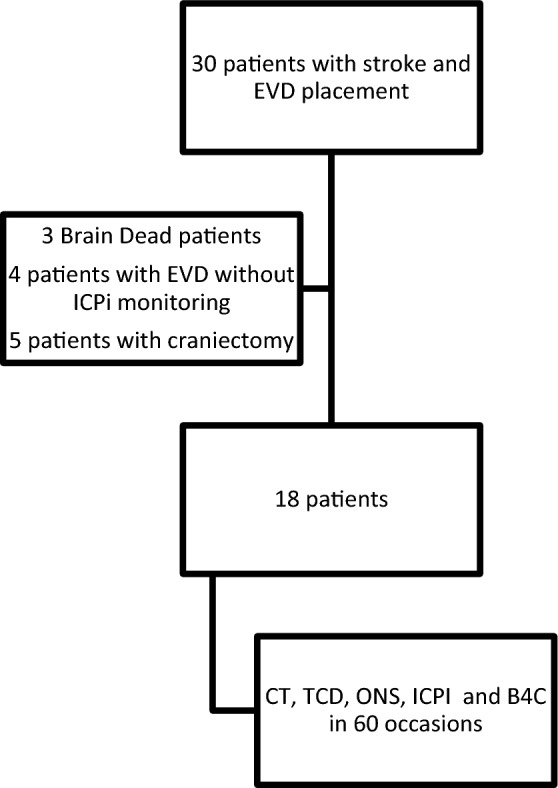
Study Flowchart. Abbreviations: B4C: Brain4care monitor; EVD: external ventricular Drainage; ICPi: Invasive Intracranial Pressure monitor. CT: Computerized Tomography; ONSD: Optic Nerve Sheath Diameter; PI: Pulsatility Index; TCD: Transcranial Doppler.

Of the 30 patients with invasive ICP monitoring admitted to our Neuro-ICU between March 2019 and March 2020 (pre-COVID-19), 18 met our inclusion and exclusion criteria (Fig. 2), with a total of 60 monitoring sessions regarding all methods described in this study. Fifty-one monitoring sessions were from patients with subarachnoid hemorrhage (SAH), two from patients with IS, and seven from patients with intraparenchymal hemorrhage (IPH). SAH etiology was 100% aneurysmal, IPH was 100% hypertensive, and the two IS were cardioembolic. Table 2 shows the clinical characteristics of the patients.

**Table 2:** Patient’s clinical characteristics (*N* = 60 measurements in 18 patients).

	Mean	Median	SD*	Minimum	Maximum
Age	53.6	54.0	14.3	31	84
NIHSS	17.4	17.0	5.67	6	28
MAP	98.5	95.0	17.0	67	140
Temperature °C /°F	36/96.9	36.2/97.1	0.585/33	35.0/95.0	38.0/100.4
Glucose	103	101	20.4	70	185
ICP minimum	9.82	6.50	11.0	0	46
ICP maximum	16.5	12.5	14.5	4	52
Hemoglobin	10.8	11.0	1.06	8.80	13.6

°C: Celsius; °F: Fahrenheit; *ICP*: Intracranial Pressure; *NIHSS*: National Institute of Health Stroke Scale; *MAP*: Mean Arterial Pressure.

### ICP wave morphology—Brain4care method

The mean time of invasive and noninvasive monitoring with Brain4Care (B4c) was 45 min (35–60 min), with a mean of 3.290 waves per monitoring (IC 2.080–4.310), totaling 197.400 B4c waves distributed in 2.495 min. In 30% (18 out of 60) of the monitoring sessions, the mean ICP was higher than 20 mmHg. The morphological characteristics of B4c waves are described in Table 3.

**Table 3:** Morphological characteristics of the ICP waves.

	P2P1 B4C	TTP B4C
Minutes	2495	2495
Mean	1.17	0.219
Median	1.18	0.215
Standard deviation	0.326	0.104
Minimum	0.420	0.0370
Maximum	2.93	0.820

*B4C*: Brain4care monitor; P2/P1: P2/P1 ratio; *TTP*: Time-to-Peak.

**Figure 3:**
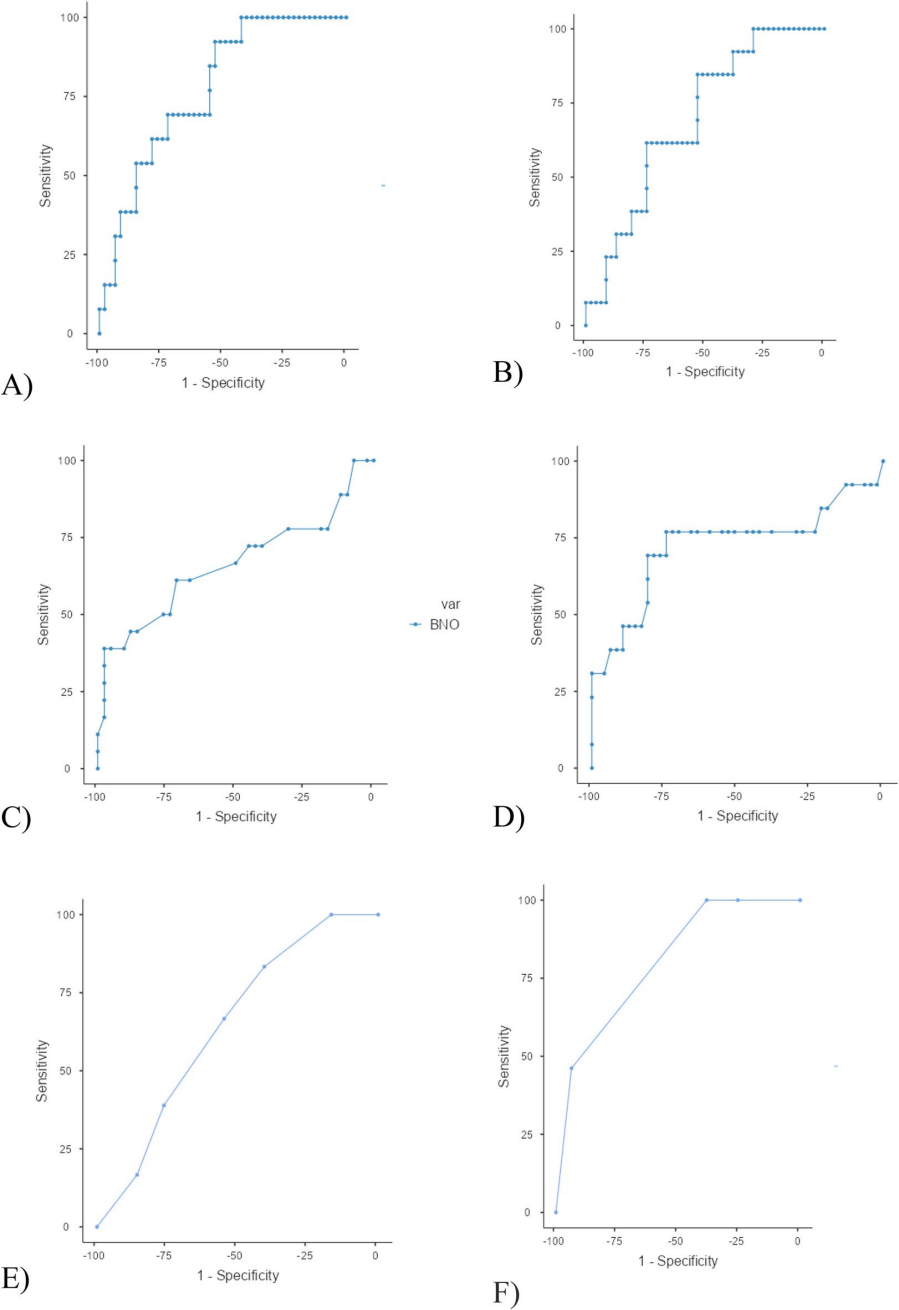
ROC curves of the noninvasive methods. (**A**) mean P2/P1 (**B**) mean TTP. (**C**) mean ONSD D) PI of TCD, (**E**) Sum of CT, (**F**) Combination of B4c and TCD. Abbreviations: B4C: Brain4care monitor; CT: Computerized Tomography; P2/P1: P2/P1 ratio; ONSD: Optic Nerve Sheath Diameter; PI: Pulsatility Index, TTP: Time-to-Peak; TCD: Transcranial Doppler.

The mean B4c P2/P1 ratio correlated with the absolute ICP value (*r* = 0.35, *p* < 0.001). The correlation between the mean TTP and the mean ICP was also significant (*r* = 0.35, *p* < 0.001). The mean B4c P2/P1 ratio showed a sensitivity of 100%, specificity of 45.5%, positive predictive value (PPV) of 36.8%, and negative predictive value (NPV) of 100% to predict ICH, with a cutoff value of 1.06. TTP showed a sensitivity of 85.7%, specificity of 50.0%, PPV of 35.3%, and NPV of 91.7% to predict ICH, with a cutoff value of 0.199. The AUC was 0.79 (Fig. 3A) for the mean P2/P1 and 0.69 for the mean TTP to predict ICH. (Fig. 3B).

### Optic nerve sheath Ultrasonography

A total of 360 measurements were performed on 60 separate monitoring sessions. The mean ONSD was 4.93 mm (CI 3.57–7.70). The mean ONSD was 5.69 (CI 4.82–6.42) in sessions in which patients had ICH and 4.55 (CI 4.33–4.77) mm in sessions in which ICP was below 20 mmHg (p = 0.006). The mean ONSD difference between sides was 0.06 mm. The intraclass correlation coefficient (ICC) for assessing agreement between the right and left eye was high (0.92, CI 95% 0.87–0.96), suggesting a small side-to-side variation. The correlation between the mean ONSD value and the mean ICP was weak (*r* = 0.29, p = 0.01). The AUC for diagnosing ICH was 0.69 for the mean ONSD. With a cutoff of 5.2 mm, the sensitivity was 71.4%, the specificity was 70.4%, the PPV was 43.5%, and the NPV was 88.6% (Fig. 3C).

### Transcranial Doppler

The 18 patients underwent 60 monitoring sessions, with 120 PI measurements (one measurement on each side). The mean PI on both sides was 1.22 (minimum 0.785, maximum 3.33). The mean difference between sides in the same patient was 0.12 (CI95% 0.08–0.16). The ICC for assessing agreement between the right and left PI was high (0.88, CI95% 0.83–0.91), suggesting slight side-to-side variation. The mean PI in patients with and without ICH was 1.09 and 1.45, respectively (p = 0.002). The correlation between mean PI and mean ICP was moderate (*r* = 0.62, *p* < 0.001). The mean PI showed an AUC of 0.75 to diagnose ICH. With a PI threshold of 1.28, the sensitivity was 78.6%, the specificity was 77.3%, the PPV was 50.0%, and the NPV was 91. 7%. (Fig. 3D). We performed a sensitivity analysis using only the time that overlapped between TCD and the B4c and found consistent results.

### Brain CT scan

Overall, 55 of 60 (91.6%) CTs showed at least one radiological sign suggestive of ICH. CTs in which patients did not have ICH showed a median of two findings (minimum 0 and maximum 3), and a median of three findings (minimum 2 and maximum 5) when patients had ICH (p = 0.05). The correlation between the sum of CT findings and the mean ICP was weak (*r* = 0.21, p = 0.03). The AUC of the ROC curve of the sum of CT for ICH was 0.644 (Fig. 3E). Finding a signal on CT showed a sensitivity of 100%, a specificity of 16.6%, a PPV of 34%, and a NPV of 100% for detection of ICH.

### Summary of the findings of the individual methods

Table 4 summarizes the correlation between noninvasive methods and ICP and the AUC values of the ROC curve with a defined cutoff value.

**Table 4:** Summary of the noninvasive methods and their correlation with ICP, ROC AUC, and the cutoff point for each method.

Noninvasive method	ICP correlation	AUC (ROC)	Cutoff point
Mean P2/P1 B4C	0.35	0.786	1.06
Mean TTP B4C	0.35	0.694	0.2
Mean ONSD	0.29	0.692	5.2
Mean PI TCD	0.62	0.750	1.28
Sum CT	0.21	0.644	2

*B4C*: Brain4care monitor; *CT*: Computerized Tomography; P2/P1: P2/P1 ratio; *ONSD*: Optic Nerve Sheath Diameter; *PI*: Pulsatility Index, *TTP*: Time-to-Peak, *TCD*: Transcranial Doppler.

### Logistic regression model for repeated measures

After multivariate analysis, only three of the variables described remained statistically significant: Mean B4C TTP, mean PI, and mean optic nerve sheath** (**ONS). The ROC curve of the sum of the three methods had an AUC of 0.861. The details are presented in Table 5. Comparing the aggregate accuracy of only two methods at a time the best combination (pair) of methods was B4c and TCD, with a ROC curve with an AUC of 0.802 (Fig. 3F).

**Table 5:** Description of the multivariate GEE findings for Intracranial Hypertension.

Name	OR	95% CI (minimum)	95% CI (maximum)	p
Mean TTP B4C	36.206	26.302	50.486	0.008
Mean PI TCD	3.875	1.543	6.497	0.025
Mean ONSD	1.074	1.009	1.144	0.026

*B4C*: Brain4care monitor; *IH*: Intracranial Hypertension, *ONSD*: Optic Nerve Sheath Diameter; *PI*: Pulsatility Index, *TTP*: Time-to-Peak, *TCD*: Transcranial Doppler.

## Discussion

We found that the noninvasive methods used to evaluate the presence of ICH showed a wide range of correlation with ICP, from weak to moderate. Conversely, the discriminatory power of all four methods was moderate to high. When combined, B4c and TCD showed the best diagnostic ability.

The B4C wave morphology showed adequate performance, with a moderate correlation with ICP. The parameter P2/P1 was better than TTP in predicting ICH in the univariate analysis, although only TTP remained significant in the multivariate analysis. It is known that morphological changes in the ICP waveform precedes ICH^18–20^. Consequently, P2/P1 and TTP could be altered even before the onset of ICH depending on the intracranial compliance^19^**.** This could explain, at least in part, why some patients had abnormal P2/P1 and TTP but normal ICP value, justifying physiologically its moderate correlation to the ICP value and to predict ICH. This hypothesis is supported by a study that studied 38 patients with traumatic brain injury (TBI) and found an abnormal P2/P1 ratio in several patients with no ICH^21^. Another recent study analyzed the association between noninvasive wave morphology and intracranial compliance (ICC)^22^. A total of 36 patients with ICPi monitoring underwent an intracranial volume infusion test while monitored with TCD. Three methods were used to evaluate the ICC: a) a mathematical fluid model, b) an assessment of cerebral arterial blood volume changes, and c) the ratio of the amplitude of pulse wave P2/P1 peaks. All methods showed a strong and positive correlation with ICC. The P2/P1 ratio showed a correlation of 0.94 with ICC. Another recent study by Ballestero et al.^23^ aiming to assess the ICP waveform parameters on their ability to predict IH and functional prognosis evaluated 22 TBI patients simultaneously with B4C sensor and ICPi. The findings unveiled a robust non-linear correlation between ICPi and non-invasively derived nICPW waveforms, despite the presence of a moderate linear Pearson's correlation. However, the non-invasive parameters did not exhibit any association with patient outcomes or IH. This study used extended monitoring periods, a concept that, while interesting, may introduce certain limitations^24^. Considering the sensor's mechanical nature and its direct contact with the skull's surface, continuous monitoring in the context of an intensive care unit's daily operations could be susceptible to disruptions caused by patient movement or handling. Such factors could potentially undermine the precision of the assessments. In contrast, our study opted for a briefer monitoring duration, accompanied by vigilant oversight. This approach aligned more effectively with the device's current capabilities^15^.

In our study, ONSD showed worse results than expected compared to the previous literature. The initial studies^25^ showed a cutoff of 4.8 mm, with a sensitivity of 96%, specificity of 94%, and AUC = 0.98. Several other investigators corroborated these initial findings with cutoff points ranging from 4.8 to 5.2 mm^26–29^. A meta-analysis^30^ confirms that ONSD > 5.00 mm presents a sensitivity of 99% and specificity of 73%, including six studies with 352 patients with an AUC of 0.981. These encouraging results have been challenged by other investigators, who found a very weak correlation between the ONSD and the ICPi in patients with SAH^31,32^. A possible loss of the elastic properties of the ONS acutely distended due to very high ICP during aneurysmal rupture might be a possible explanation for the poor correlation of ICP and ONSD in patients with SAH. Hansen et. al explored the elastic properties of the ONS using human optic nerve preparations isolated from autopsies and used progressive pressure increases to show that moderate ICP increases allowed reversible sheath changes. However, when the pressure increase was equal to or greater than 45 mmHg, there was hysteresis, i.e. no return to the baseline diameter^33^. This is clearly relevant in SAH since ICP may rise to 100 mmHg during aneurysmal rupture both in humans and in experimental models, which is one of the reasons for the initial loss of consciousness some patients experience^34,35^.

The literature using TCD to predict ICH can be divided into three groups: predictions based on the PI, those based on the estimation of noninvasive cerebral perfusion pressure, and those using mathematical models. The published studies showed an accuracy for ICH prediction ranging from 0.62 to 0.92 (AUC)^36^. Bellner et al. evaluated 82 patients with distinct etiologies (SAH, TBI, and others) and reported an excellent ICC with ICP (R^2^ = 0.938) with the formula: ICP: 10.93 X PI—1.28. No other study has been able to replicate their results with such accuracy^37–41^. In our study, TCD showed the best correlation with ICP (*r* = 0.62) and the second-best ROC curve, with an AUC of 0.75, when corrected for its classic confounding variables such as vasospasm, fever, and anemia, keeping its significance after the multivariate analysis. Recently, a prospective, international, multicenter, unblinded, diagnostic accuracy study recruited two hundred and sixty-two patients in 16 international intensive care units. ICP*i* measurements (reference test) were compared to simultaneous ICP*tcd* measurements^42^ *(*index test) at three different timepoints: before, immediately after and 2 to 3 h following ICP*i* catheter insertion. The results showed an elevated NPV (ICP > 20 mmHg = 91.3%, > 22 mmHg = 95.6%, > 25 mmHg = 98.6%), indicating high discriminant accuracy of ICP*tcd* in excluding IH^43^.

In our study, we used the only CT scale created for ICH detection, which was developed in the original study by Miller et al.^44^ and later adapted by Rajajee et al.^14^ (Table 1). Miller evaluated 160 patients with Traumatic Brain Injury using ICPi monitoring to investigate if the five criteria observed predicted ICH. Although the binary logistic regression showed no significance, all factors were statistically significant in the linear regression. Based on this previous study, Rajajee et al. evaluated 65 patients diagnosed with SAH, TBI, intraparenchymal HS, and tumor. As in our study, most patients had at least one of the described signs (91.6% in our study and 77% in the cited study) related to ICH. The sensitivity of the original study to detect ICH with at least one radiological sign was 77% (CI95% 46–95%), specificity was 29% (17–45%), PPV was 24% (CI95% 12–40%), and NPV 81% (CI95% 54–96%). Such findings corroborate our results, with high sensitivity and NPV and low specificity and PPV for ICH prediction.

Only one previous publication evaluated the ability of multiple noninvasive methods to estimate ICH^7^. A total of 100 patients were evaluated (TBI = 30; SAH = 47; HS = 23) with invasive monitoring associated with ONSD, TCD (PI and ICP estimation formula [eICP]), and Pupillary Neurological Index (NPI) by automated pupillometry. All four methods showed moderate correlation with ICPi (ONSD, *r* = 0.54; PI, *r* = 0.50; eICP, *r* = 0.61; NPI, *r* =  − 0.41, with *p* < 0.001 for all methods). The AUC to predict ICH was 0.78 for ONSD, 0.85 for PI, 0.86 for eICP, and 0.71 for NPI. With the combination of techniques, the highest AUC was 0.91 (0.84–0.97) with the combination of ONS and eICP. In a subgroup analysis, patients with SAH showed higher correlation with pupillometry (*r* = -0.77) and eICP (*r* = 0.88), and moderate correlation with ONS (*r* = 0.61) and PI (*r* = 0.53). The AUC to predict ICH in this subgroup was 0.78 for ONSUS, 0.79 for PI, 0.68 for eICP, and 0.70 for NPI. This study corroborates the concept that noninvasive techniques exhibit a satisfactory ICP correlation and a reasonable predictive accuracy for ICH particularly enhanced when multiple methods are employed in concert. In the whole group of patients, TCD and ONSD was the best combination, while in the SAH subgroup, NPI and TCD showed the best performance for ICH prediction. In our study, the combination of TCD and B4C had the best accuracy. We acknowledge the existence and importance of other relevant noninvasive methods besides the ones discussed in this paper and further discussion can be found elsewhere^2,5,6^.

Our study presents some limitations. First, it is a single-center study with few subjects (*n* = 18), although the number of measurements was reasonable (*n* = 60). Second, the time between admission and assessment varied among patients, as well as the interval between measurements in the same patient and among patients. Third, PaCO_2_ was not universally monitored as a confounding variable. Furthermore, the ONSD and TCD examiners were not blinded to the ICP measures. Finally, the correlations we observed between the various methods and ICP were characterized by modest to moderate strengths. This pattern is likely attributable to each method gauging distinct surrogates or proxies of intracranial hypertension, rather than directly quantifying ICP itself.

## Conclusion

In conclusion, this exploratory study with noninvasive methods showed various degrees of correlation with ICP and discriminatory power for the diagnosis of ICH. The noninvasive wave morphology monitor and TCD (PI) had the best performances both isolated and combined. The multimodal combination of such indices may increase the ability to noninvasively identify and manage ICH. The observed diversity in estimated ICP through non-invasive measurements underscores the need for additional investigations to define the appropriateness of specific methods more precisely within distinct clinical scenarios.

## Data Availability

The datasets used and/or analyzed during the current study are available from the corresponding author on reasonable request.

## Abbreviations

ICH: Intracranial hypertension
SAH: Subarachnoid hemorrhage
IPH: Intraparenchymal hemorrhage
IS: Ischemic stroke
ICPi: Invasive intracranial pressure
ONS: Optic nerve sheath
ONSD: Optic nerve sheath diameter
ONSUS: Optic nerve sheath ultrasound
PI: Pulsatility index
TCD: Transcranial Doppler
CT: Computed tomography
TTP: Time-to-peak
B4c: Brain4Care
ICP: Intracranial pressure
ICPni: Noninvasive intracranial pressure method
HS: Hemorrhagic stroke
neuro-ICU: Neurocritical care unit
EVD: External ventricular drainage
MCA: Middle cerebral artery
ICC: Intraclass correlation coefficient
ROC: Receiver operator characteristic
AUC: Area under the curve
OR: Odds ratio
CC: Cerebral compliance
TBI: Traumatic brain injury
ICC: Intracranial compliance
